# HIV in Maternal and Child Heath: Concurrent Crises Demand Cooperation

**DOI:** 10.1371/journal.pmed.1000311

**Published:** 2010-07-27

**Authors:** The PLoS Medicine Editors

## Abstract

The *PLoS Medicine* editors argue that the time has come to integrate prevention and treatment of HIV into maternal and child health care programs.

In June, *PLoS Medicine* published a series of articles on interventions to tackle the burden of maternal, neonatal, and child mortality in sub-Saharan Africa and how to implement those interventions most effectively [1]–[6].

Reducing child mortality and improving maternal health are two of eight Millennium Development Goals (MDGs) agreed to by 191 UN member states. The lack of progress on these goals so far means that the 2015 targets for maternal and child health are unlikely to be met. In the midst of a flurry of conferences (World Health Assembly, Women Deliver 2010, Countdown to 2015 in 2010, Global Health and Global Metrics, Pacific Health Summit, and the G8 meeting in Canada) in which politicians, scientists, and public health specialists are assessing global MDG progress, one questions the extent to which these issues can ever be addressed by considering each as separate from the others. The need to adopt a more integrated approach to global health problems is starkly illustrated by the confluence of HIV/AIDS and maternal health: A recent, updated analysis of maternal deaths worldwide revealed that without the HIV epidemic there would have been an estimated 61,400 fewer maternal deaths in 2008 [7].

Indeed, Robert Black and colleagues, writing on maternal, neonatal, and child health in Africa in the *PLoS Medicine* series, point out a need to identify and address specific infectious disease problems as part of a stepwise process toward strengthening health systems [2]. They cite a previous study by Joy Lawn and colleagues in South Africa that reported approximately 300,000 mothers with HIV/AIDS giving birth every year, with the toll of HIV infection contributing to 57% of all child deaths. According to the same article, 7.2% of all 6-week-old infants attending their first immunization were already HIV-infected, despite a national program launched in 2000 to provide single-dose nevirapine to HIV-positive women in labor and to their infants postnatally. If South Africa were to scale up interventions for prevention of mother-to-child transmission of HIV (PMTCT) with appropriate feeding choices to cover 95% of mothers and newborns, more than 37,000 children could be saved each year, say the authors [8].

Although tackling HIV is a priority within the MDGs, it is linked to other infectious diseases—namely malaria and tuberculosis— and is not specifically included in the MDGs for maternal and child health. While it is clearly crucial to reduce the global burden of infectious diseases, the inclusion of HIV as only part of an infectious disease–specific health goal isolates HIV infection from the population of vulnerable women and children it affects. A stronger recognition of the linkages between HIV and maternal and child health is necessary to improve the health of these affected populations. In an example of how this can be done, last year WHO and UNICEF issued a joint statement calling for routine home visits for all newborn babies in their first week of life, especially for low–birth-weight babies and for HIV-positive women and children [9]. New WHO guidelines on PMTCT [10] emphasize the need to tackle HIV in the context of maternal and neonatal health. However, according to the HIV/AIDS charity Avert, issues surrounding implementation of PMTCT are complex and are closely linked with the stigma surrounding HIV infection. Of particular concern is the need to reach women in need of PMTCT and the provision of suitable guidance on key issues such as infant feeding. The reasons women may not access PMTCT need to be investigated more thoroughly, but they include not being offered an HIV test or refusing to take an HIV test, failure to return after a test for follow-up visits, and not adhering to self-administered drug regimens [11].

It is clear that tackling HIV as it affects all populations should not eclipse other health problems, but governments, funders, and NGOs should consider maternal and child health with the same urgency as they have the global HIV crisis. Concurrent crises should provide opportunities for synergy in global health programs, rather than competition for attention and resources. During meetings in April of this year, the Global Fund recognized the interconnectedness of maternal and child health with their core mandate around HIV, tuberculosis, and malaria, and on 20 May 2010 called for new projects to link the health of women and children with disease-specific initiatives to produce a cross-cutting agenda [12]. They recommended “integrated approaches to achieve the health-related MDGs and improve health outcomes for women and children” [13]. Such approaches might include HIV, tuberculosis, and malaria interventions that are applied throughout the continuum of prepregnancy, pregnancy, birth, and childhood, and training of local health workers to promote women's access to information related to HIV and sexual and reproductive health. These types of approaches would integrate rather than separate HIV from maternal and child health.

The July issue of *PLoS Medicine* also features two magazine articles that further highlight the importance of tackling HIV in the context of women's and child health. Agnès Binagwaho and colleagues provide an update on the Rwanda Learning Collaborative on Child Health, which aimed to increase access to and quality of PMTCT services in the Eastern Province of Rwanda using a collaborative learning model [14]. According to the authors this quality improvement initiative provides an organizing tool to mediate change with government and NGO support. Also in this issue, Scott Kellerman and Shaffiq Essajee examine different mechanisms for HIV testing in children, arguing that a focus on child testing apart from PMTCT is long overdue [15].

Change is afoot. In his opening address to the delegates at the Women Deliver 2010 conference, Michel Sidibé, UNAIDS Executive Director, said that “If we integrate HIV into maternal health programs, we can make huge progress on almost every global development goal. We can stop mothers from dying of HIV and dramatically reduce maternal mortality. Let's join together.” [16]. At the same conference, Melinda Gates pledged US$1.5 billion over the next five years to stimulate investment into integrated women's and child health programs [17]. And, ahead of the G8 conference in Canada, UNAIDS Deputy Executive Director for Management and External Relations Jan Beagle called for an integrated approach bringing together MDGs 4, 5, and 6 and said that “As HIV is the leading cause of death among women of reproductive age, the global response to AIDS can and must be leveraged more effectively to meet women's health needs” [18]. It seems that the time has come to stop tackling infectious diseases, specifically HIV, in isolation from other health goals, and instead to examine the opportunities for integrating and scaling up delivery of HIV prevention and treatment services within the context of maternal and child health care.

## Abbreviations

MDG: Millennium Development Goal
PMTCT: prevention of mother-to-child transmission
